# Editorial: Impact of Lipid Peroxidation on the Physiology and Pathophysiology of Cell Membranes

**DOI:** 10.3389/fphys.2016.00423

**Published:** 2016-09-22

**Authors:** Angel Catalá, Mario Díaz

**Affiliations:** ^1^Facultad de Ciencias Exactas, Instituto de Investigaciones Fisicoquímicas Teóricas y Aplicadas (INIFTA-CCT La Plata-CONICET), Universidad Nacional de La PlataLa Plata, Argentina; ^2^Departamento de Biología Animal, Edafología y Geología, Facultad de Ciencias, Sección Biología, Universidad de La LagunaTenerife, Spain; ^3^Unidad Asociada de Investigación CSIC-ULL “Fisiología y Biofísica de la Membrana Celular en Patologías Neurodegenerativas y Tumorales”Tenerife, Spain

**Keywords:** lipid peroxidation, cell membranes

Oxygen-derived free radicals such as hydroxyl and hydroperoxyl species have been demonstrated to oxidize membrane lipid components, mainly phospholipids, leading to lipid peroxidation. Membrane phospholipids containing polyunsaturated fatty acids are predominantly susceptible to peroxidation. Free radical catalyzed peroxidation of long chain polyunsaturated acids (LCPUFAs) such as arachidonic acid and docosahexaenoic acid leads to generation of LCPUFA metabolites, including endoperoxides, isoprostanes, and neuroprostanes, which may further exert pharmacological/toxicological actions in many tissues (Balazy, 2000; Greco et al., 2000; Hardy et al., 2005; Bochkov et al., 2010; Davies and Guo, 2014). Eventually, peroxidation of membrane lipids can disturb the assembly of cell membranes, which inevitably will impact membrane fluidity, lipid-lipid, and lipid-protein interaction dynamics, membrane permeability, physicochemical properties, ion, and nutrient transport, membrane-initiated signaling pathways, and metabolic processes leading to cell death (Fruhwirth and Hermetter, 2008; Catalá, 2009; Adibhatla and Hatcher, 2010; Volinsky and Kinnunen, 2013). Intensive research performed over the last decades have also revealed that products of lipid peroxidation are also involved in cellular signaling and transduction pathways under physiological conditions, and regulate a variety of cellular functions, including normal aging (Bazan, 2005; Pamplona, 2008; Naudí et al.).

The present research topic might be divided in two major blocks. The first one deals on the mechanisms of lipid peroxidation under physiological and pathophysiological conditions. The second one highlight the different defenses against lipid peroxidation and oxidative stress that cells contain and the efficient and regulated mechanisms they use to buffer the noxious effects of lipid peroxidation. In the next paragraphs, we will summarize the highlights of each of these blocks and the articles included therein.

In the first block, Njie-Mbye et al. review the pathophysiological and pharmacological implications of lipid peroxidation in the eye. The eye, and particularly the retina, is the organ containing the largest amount of LCPUFAs amongst all organs in the body. Further, lipid peroxidation has been reported to be involved in degenerative ocular diseases, such as age-related macular degeneration, cataract, glaucoma, and diabetic retinopathy. Authors review the toxicological effects of hydrogen peroxide, LCPUFA-derived lipoperoxides and several synthetic peroxides in different parts of mammalian eyes, including the uvea, ganglionic retinal cells, and posterior segments in the retina. They also show that H_2_O_2_ and LCPUFA-derived lipoperoxides modulate excitatory and inhibitory synapses in the eye by regulating neurotransmitter metabolism, and conclude that these abilities to alter the integrity of neurotransmitter pools provide new potential target sites and point to novel pharmacological strategies for the treatment of degenerative ocular diseases.

Lipid peroxidation of biological membranes modifies their assembly, structure, and dynamics, both at lipid and protein levels. When the Fluid Mosaic Model (FMM) of membrane structure was introduced by Singer and Nicolson (1972), it was visualized as a basic model for cell membranes that could explain existing observations on membrane proteins and lipid structures and their dynamics. Accordingly, the membrane was topologically defined as a biological fluid of proteins and lipids oriented in two dimensions. In the FMM, amphipathic phospholipids are oriented in a lamellar mesophase organization with hydrophobic fatty acyl chains embedded within the interior of the membrane and the hydrophilic polar groups facing the aqueous environment. However, several biological processes cannot be explained on the basis of this typical phospholipid orientation but exhibit other conformations. The Lipid Whisker Model (LWM) is an extension of the FMM, introduced upon observations into the conformation of oxidized phospholipid (oxPL) species recognized by CD36 (Li et al., 2007). Thus, in the LWM, when cell membranes undergo oxidation, if not adapted by the action of phospholipases, they may “produce whiskers” including a variety of oxidized sn-2 fatty acids of diverse structures. In the (LWM), the assembly of many oxPL within cell membranes is different compared with one observed in non-oxPL described in the FMM. Indeed, biophysical evidence indicates that addition of an oxygen atom to the acyl chain produces significant changes that prevent its immersion in the interior of the membrane, because the presence of peroxyl radicals project toward the aqueous interface. However, controversies exist on the validity of the validity of this “floating peroxyl radical” hypothesis. This has been discussed in the opinion article by Dr. Catalá.

The article by Pizzimenti et al. revise the interaction of membrane proteins and aldehydes derived from lipid peroxidation under both, physiological and pathophysiological perspectives. They begin their review with a description of main aldehydes (4-HNE, acrolein, MDA, and Phosphatidyl γ-Hydroxyalkenals) protein adducts. They show that targets of lipid peroxidation-derived aldehydes are cell-type specific and dependent on both, the set of proteins expressed in the specific cell type and also by the concentration of reactive aldehydes. Not all protein-aldehyde adducts are necessarily deleterious, and the final biological effect will depend on the adduct concentration. For instance, at low concentration, HNE have an important role in signal transduction pathways, and exert anti-proliferative and anti-metastatic effects toward cancer cells, by interfering with the modulation of gene expression through generation of protein and/or DNA adducts. Under a pathological scenario, Pizzimenti and collaborators review the main evidences linking these protein-adducts with chronic neurodegenerative diseases (Alzheimer's disease), atherosclerosis and autoimmunity inflammation. In line with this, a methodological article by Wu et al. describe and validate a novel two-dimensional native SDS-PAGE that allows identification of HNE-protein adducts. The example illustrated in the article show the procedure applied to identify mitochondrial complex I (NADH-ubiquinone oxidoreductase), which are linked to diabetic kidney mitochondria.

Membrane lipid peroxidation is not limited to the generation of reactive oxygen species, but also to the production of nitrogen reactive species (RNS) from nitric oxide (NO). NO is recognized both, as a signaling molecule that regulates many enzyme activities, but also as a toxic agent in animal and plant cells. NO is able to protect photosynthetic and non-photosinthetic organims from oxidative damage resulting from O^·−^, H_2_O_2_, and alkyl peroxides by acting as a terminator of free radical chain reactions. Through the reaction of O-2 with NO, the anion peroxynitrite (ONOO^−^) is generated, which acts as a reactive nitrating agent capable of modifying proteins (nitrotyrosine), lipids (lipid nitration), and nucleic acids (DNA nitration) (Gisone et al., 2003). These reactive effects of RNS on lipid peroxidation are commented in the article by Galatro and co-workers in the present Research Topic (Galatro et al.).

The second block of articles included this research topics are introduced by a review article by Naudí et al. related to the involvement of membrane lipid unsaturation as a physiological adaptation to animal longevity. After introducing the dual role of lipid peroxidation-derived metabolites as cell threatening or signaling molecules, authors use a comprehensive comparative study of animals along the phylogenetic tree to postulate that longevity is associated to membrane lipid unsaturation. Through a series of targeted global queries they speculate and expand on (1) The link between aging, membrane unsaturation and lipoxidation reactions, (2) Whether interspecies variations in longevity are related to differences in membrane unsaturation and lipoxidation-derived molecular damage, and (3) Whether genetic manipulations or nutritional interventions might be accompanied by attenuations of membrane unsaturation in cell membranes. Finally, authors provide a hypothesis to describe the physiological mechanisms underlying the structural adaptation of cellular membranes to oxidative stress and to review the state of the art about the link between membrane composition and longevity of animal species.

The last three articles are focused on bioactive molecules participating in cellular antioxidant strategies. The first, from the group of Dr. Reiter in Texas, is an opinion article on the role of melatonin and its metabolites, such as cyclic 3-hydroxymelatonin or N1-acetyl-N2-formyl-5-methoxykynuramine, in scavenging lipoperoxides (by acting as a direct scavenger of initiation and propagation by-products) and also on their ability to modulate the activities of a variety of antioxidant enzymes (Reiter et al.). These effects are physiologically relevant, since melatonin is produced in many cells and its synthesis may be upregulated under conditions that elevate oxidative stress in mammals, where they have been proven to exert protective actions both *in vivo* and *in vitro* and in models of numerous diseases.

Casañas-Sánchez and co-workers contribute to this topic with two articles on the mechanistic basis of action of two novel “*indirect antioxidants*” in model hippocampal cells. The term “*indirect antioxidants*” was introduced in recent years to refer to bioactive molecules that although lacking direct scavenging activities, they stimulate antioxidant activity by modulating gene expression of components of the antioxidant systems. In the first study, a hypothesis and theory, they show that docosahexaenoic acid (DHA) is, besides a structural component of membrane phospholipids a efficient activator of specific genes belonging to the glutathione/glutaredoxin and thioredoxin/peroxiredoxin systems (Casañas-Sánchez et al.). Their hypotheses focus on the ability of DHA to regulate the expression of phospholipid-hydroperoxide glutathione peroxidase (PH-GPx/GPx4) isoforms, the main enzyme family protecting cell membranes against lipid peroxidation and capable to reduce oxidized phospholipids *in situ*, i.e., within the membrane. By doing this, DHA triggers the stimulation of genomic mechanisms leading to self-protection from oxidative damage. This is particularly relevant in neuronal cells, where the high aerobic metabolism and the presence of elevated levels of transition metals, favor the generation of reactive oxygen species and lipid peroxides from highly unsaturated fatty acids, as is the case of DHA and arachidonic acid. Authors also show that, at least in mouse hippocampal neurons (cell lines and hippocampal parenchyma), DHA upregulates a Gpx4 splicing variant (named Gpx4 CIRT), which harbors part of the first intronic region (CIRT: Cytoplasmic Intron-sequence Retaining Transcripts), which according to the “sentinel RNA hypothesis” (Buckley et al., 2014) would expand the ability of Gpx4 family of transcripts to provide neuronal antioxidant defense in cellular compartments, like dendritic zones, located away from the nucleus, independently of conventional nuclear splicing.

The second article by Casañas-Sánchez et al. is rather striking, since they show that ethanol may well be neuroprotective to hippocampal cells. Ethanol is known to cause severe systemic damage secondary to oxidative stress, and brain is particularly vulnerable to ethanol-induced reactive oxygen species, especially upon high and/or chronic exposures to ethanol. However, Casañas-Sánchez et al. demonstrate that a low and acute dose of ethanol trigger a completely opposite response in hippocampal cells, consistent with the stimulation of transcriptional expression of genes belonging to the classical, glutathione/glutaredoxin, and thioredoxin/peroxiredoxin antioxidant systems. That these genomic effects are neuroprotective is demonstrated in experiments where they show that sub-toxic ethanol exposure prevents glutamate-induced excitotoxicity in hippocampal cells, pinpointing that under this paradigm ethanol may well be neuroprotective against oxidative insults in hippocampal cells.

Overall, we believe our Research Topic will provide an exciting overview on the impact of membrane lipid peroxidation under physiological and pathophysiological scenarios. Currently, there exists an increasing appreciation on the importance of membrane lipid peroxidation in relation to health and disease, as well as longevity. We hope that this Research Topic will foster the research on the precise involvement of membrane lipid peroxidation in devastating diseases such as neurodegenerative and cardiovascular diseases, as wide spread disorders in developed societies, and on the development of pharmaceutical strategies aimed to prevent and treat them.

## Author contributions

AC and MD have edited the Resarch Topic and have written and drafted the manuscript.

### Conflict of interest statement

The authors declare that the research was conducted in the absence of any commercial or financial relationships that could be construed as a potential conflict of interest.
